# Development of a Taqman Real-Time PCR for the Identification of *Haemaphysalis longicornis* (Acari: Ixodidae)

**DOI:** 10.1093/jme/tjac074

**Published:** 2022-05-28

**Authors:** Guang Xu, Fumiko Ribbe, Joseph McCaffery, Chu-Yuan Luo, Andrew Y Li, Stephen M Rich

**Affiliations:** Department of Microbiology, University of Massachusetts, Amherst MA, USA; Department of Microbiology, University of Massachusetts, Amherst, MA, USA; Department of Microbiology, University of Massachusetts, Amherst, MA, USA; Department of Microbiology, University of Massachusetts, Amherst, MA, USA; Invasive Insect Biocontrol and Behavior Laboratory, USDA-ARS, Beltsville, MD, USA; Department of Microbiology, University of Massachusetts, Amherst, MA, USA

**Keywords:** Asian longhorned tick, *Haemaphysalis longicornis*, real-time PCR, pathogen

## Abstract

*Haemaphysalis longicornis* Neumann, a vector of various pathogens with medical and veterinary importance, is a recent invasive species in the United States. Like many tick species, discerning *H. longicornis* from congeners can be a challenge. To overcome the difficulty of morphological identification, a Taqman quantitative real-time PCR based on the internal transcribed spacer gene (ITS2) was developed for quick and accurate identification of *H. longicornis* with a detection limit of as low as 19.8 copies. We also applied the assay to 76,004 archived ticks and found 37 ticks were *H. longicornis*. One *H. longicornis* was submitted from Warren, Somerset County, New Jersey in June 2015, 2 yr earlier than the initial report from the United States. None of these 37 *H. longicornis* was positive for *Anaplasma phagocytophilum*, *Borrelia burgdorferi sensu lato*, *B. miyamotoi*, *B. mayonii*, *Babesia microti*, or *Ehrlichia muris*–like agent.

The Asian longhorned tick, *Haemaphysalis longicornis* Neumann, is an important vector of 30 human and animal pathogens in Asia, including various species of *Anaplasma*, *Babesia*, *Borrelia*, *Rickettsia*, and viruses (such as deadly thrombocytopenia syndrome virus, SFTSV; Zhao et al. 2020). A single female *H. longicornis* can generate progeny without mating, thus resulting in massive host infestations. Heavy infestations of this tick species reduce dairy production up to 25% and damage wool clips in quantity and quality in New Zealand and Australia (Heath 2016).

*Haemaphysalis longicornis* is native to eastern Asia and was introduced to Australia on cattle from Japan in the nineteenth century and later established in New Zealand and several Pacific islands (Heath 2016). Since the first finding of *H. longicornis* on a sheep in New Jersey, United States in August 2017 (Rainey et al. 2018), this invasive species has been reported in 17 states at present. The recent appearance and spread of *H. longicornis* in the United States may represent a new and emerging disease threat to public and animal health (Tanne 2018).

Species identification of *H. longicornis* is critical for further tick surveillance, population management, and control of tick-borne diseases. However, the accuracy of species identification, often through morphological characters, can be challenging. *Haemaphysalis* is the second largest genus in the Ixodidae family, with over 160 species worldwide. In the United States, there are three additional *Haemaphysalis* species: the rabbit tick, *H. leporispalustris* and the bird tick, *H. chordeilis* are two native species; the Neotropical species *H. juxtakochi* is sporadically detected on birds migrating from Mexico (Egizi et al. 2019). These North American *Haemaphysalis* ticks are strikingly similar to *H. longicornis* morphologically. The mouthparts are the key morphological characters for distinguishing *Haemaphysalis* ticks; however, most *Haemaphysalis* ticks collected on humans or pets are in poor condition, and these features are often damaged or missing, leading to incorrect species identification, or making species identification impossible. Morphological speciation of engorged females and/or immature stages is often difficult.

Restriction fragment length polymorphisms (RFLPs) analysis of the 16S ribosomal RNA and DNA sequencing of the cytochrome c oxidase subunit I (COI) gene have been used to differentiate *H. longicornis* from the other *Haemaphysalis* species (Egizi et al. 2020, Thompson et al. 2020). However, DNA sequencing, conventional PCR assays, and RFLPs can be time-consuming and labor-intensive. The present study describes the development and validation of a Taqman real-time PCR for the rapid, sensitive, and specific detection of *H. longicornis* ticks to the species level. We also applied the assay to ticks collected from July 2006 to December 2020 through a public outreach service at the University of Massachusetts Amherst. Of 76,004 archived ticks, 37 were identified as *H. longicornis*. One *H. longicornis* was submitted from Warren, Somerset County, New Jersey in June 2015, 2 yr earlier than the previous report (Rainey et al. 2018).

## Materials and Methods

### Tick Collection and Identification

The ticks used for this study were submitted to a public tick testing program at the University of Massachusetts (Amherst, MA) from July 2006 through December 2020. Submitters were asked to provide the location and date of tick collection; age, gender, and species of the host; and attachment site of the tick on the host’s body according to the method described earlier (Xu et al. 2016).

Preliminary genus-level identification of each tick was based on published identification keys (Egizi et al. 2019). *Haemaphysalis* ticks were confirmed by amplifying and sequencing a partial tick mitochondrial 16S rRNA gene using the primers shown in Table 1 at annealing temperature 55°C.

**Table 1.** Primers and probe used in the Taqman real-time PCR assay developed for the identification of *Haemaphysalis longicornis*

Target gene	Type	Sequences (5ʹ-3ʹ)	Concentration (nM)	Reference
16S	Forward	TGCTGTAGTATTTTGACTATACAAAGG	400	Xu et al. 2018
	Reverse	ATCCTAATCCAACATCGAGGTC	400	
ITS2	Forward	TCTTTTGGGATGGATGCTGTGATG	100	This study
	Reverse	CGGATGCGACAACTCTCGTTG	400	
	Probe	CTTTCCCGCTGAGCCCGCACTTGTAAG 5ʹ Dye = HEX; 3ʹ Quencher = Iowa Black	200	
	Standard Curve	*Y* = −3.559*LOG(X) + 17.349, Eff. = 91.0%, RSq = 99.9%		

### DNA Extraction and gBlock Gene Fragment Synthesis

Nucleic acids were extracted from individual ticks using Epicentre Master Complete DNA and RNA Purification Kits (Lucigen Corporation, Middleton, WI. Cat #: MC85200), according to the manufacturer’s protocols. In brief, individual ticks were homogenized in lysis solution, digested with proteinase K, and incubated at 65°C for 15 min; then, the digested proteins were precipitated with MPC Protein Precipitation Reagent. Supernatants were transferred to a clean microcentrifuge tube. The nucleic acids were then precipitated with isopropanol and washed twice with ethanol. After drying, the pellet was resuspended in 50-µl water.

A double-stranded, sequence-verified gene fragment (gBlocks) based on the internal transcribed spacer 2 gene (ITS2 gene GenBank accession number MG721029) was ordered from Integrated DNA Technologies, Inc. (Coralville, Iowa). The gBlock was resuspended in TE buffer at 10ng/µL concentration.

### Real-Time PCR

TaqMan probe and primer sequences were designed to be specific for *H. longicornis* ITS2 gene. Assay specificity was first checked for primer and probe sequences using the BLAST program (http://blast.ncbi.nlm.nih.gov/Blast.cgi) and then was tested with genomic DNA from humans, mice (Promega, Madison, WI), soil, 18 common tick species in the United States (*Amblyomma americanum, A. cajennense*, *A. maculatum*, *Dermacentor andersoni*, *D. occidentalis*, *D. variabilis*, *H. leporispalustris*, *H. chordeilis*, *Ixodes affinis*, *I. angustus*, *I. cookei*, *I. dentatus*, *I. marxi*, *I. muris*, *I. pacificus*, *I. scapularis*, *I. spinipalpis*, and *Rhipicephalus sanguineus*), and *H. longicornis* ITS2 gBlocks.

We performed Taqman real-time PCR assays in 16-μl reaction volumes using the Brilliant III qPCR Master Mix (Agilent, La Jolla, CA) in a CFX96 Touch Real-Time PCR Detection System. The reaction contained 8-μl Master Mix, 100 nM forward primer, 400 nM reverse primer, 200 nM dual-labeled probe, 1-μl template DNA, and water up to 16 μl. An internal control was used for checking tick DNA quality (Xu et al. 2016). Water was run as a negative control for each test. The primers and probes were optimized at 100, 200, 300, and 400 nM concentrations. Cycling conditions included an initial activation of the Taq DNA polymerase at 95°C for 10 min, followed by 40 cycles: 95°C for 15 s and 60°C for 1 min.

## Results

### Assay Specificity and Sensitivity

Validity of the Taqman real-time PCR assay for the Identification of *H. longicornis* was demonstrated by successful amplification of DNA from ITS2 gene gBlocks and submitted ticks. The assay was specific to *H. longicornis*, as indicated by the absence of amplification from non-target samples, including genomic DNA from human, mouse, soil, and 18 common tick species. The optimized concentration for forward primer, reverse primer, and probe was 100, 400, and 200 nM, respectively.

**Fig. 1.**
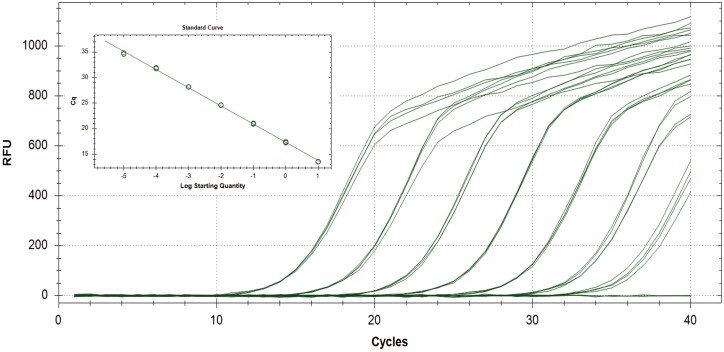
The amplification and standard curve of *Haemaphysalis longicornis* Taqman real-time PCR assay. Amplification of the tenfold dilution of ITS2 gBlock ranging from 1.984e+7 to 1.984e+1 copies. Standard curve: *Y* = −3.559*LOG(X)+17.349, Eff. = 91.0%, RSq = 99.9%.

The sensitivity of the assay was determined using a serial dilution of gBlock DNA fragments (i.e., synthetic versions of the target genes) at concentrations ranging from 1.984e+7 copies to 1.984e+1 copies per reaction. Linear regressions showed linear relationships (*r*^2^ = 0.999) between the quantities of gBlock templates and Ct values across the tested concentration range (1.984e+7 to 1.984e+2 copies). The minimum detection level was 19.8 copies of ITS2 gene. The real-time PCR efficiency was 91.0% with standard curve Y = −3.559*log(X) + 17.349 (Fig. 1).

### Testing of Submitted Ticks and Related Pathogens

Of 76,004 archived ticks from July 2006 to December 2020, 37 ticks (0.049%) were identified as *H. longicornis* using the current Taqman real-time PCR assay, including 15 larvae, 9 nymphs, 9 adults and 4 damaged ticks (unknown of life stage). The average Ct value for these 37 ticks was 17.71 (12.64 to 25.67). Among them, 1, 3, 12, 12, and 9 ticks were received from 2015, 2017, 2018, 2019, and 2020, respectively. Most (33) *H. longicornis* were received after 2018. The earliest *H. longicornis* submission was found on a “bed” from Warren, Somerset County, New Jersey in June 2015, 2 yr earlier than the previous report (Rainey et al. 2018). Twelve *H. longicornis* were found on dogs and 18 were found on humans.

We also screened for detection of common tick-borne pathogens in these ticks, as in Xu et al. (https://www.ncbi.nlm.nih.gov/pmc/articles/PMC6004831/bin/17-1755-Techapp-s1.pdf) (Xu et al. 2018). None of the 37 *H. longicornis* was positive for *Anaplasma phagocytophilum*, *Borrelia burgdorferi sensu lato*, *B. miyamotoi*, *B. mayonii*, *Babesia microti*, or *Ehrlichia muris*–like agent.

## Discussion

The primary aim of this study was to design a reliable Taqman real-time PCR method to identify *H. longicornis* ticks without the need for using morphological characters or DNA sequencing. Although RFLP analysis of the 16S rRNA and sequencing of the cytochrome c oxidase subunit I (COI) have been used to differentiate *H. longicornis* from the other *Haemaphysalis* species found in North America (Egizi et al. 2020, Thompson et al. 2020), our Taqman quantitative real-time PCR assay is rapid, sensitive, and specific for identifying *H. longicornis*. ITS2 sequences have been used for tick phylogenetic studies due to it being multicopy and highly variable (Fukunaga et al. 2000). In this study, *H. longicornis*-specific primers and probes were derived from a nonconserved region in the ITS2. The primers and probe were evaluated for possible cross-reactivity with other species of ticks and environmental samples, both in silico by sequence alignment against the current GenBank database using BLAST, and experimentally by testing the assay against DNAs from 18 common tick species. No cross-reactivity was observed including against two native species, *H. leporispalustris* and *H. chordeilis*. However, the assay was not tested using *Haemaphysalis* collections from outside the United States; thus, we cannot completely rule out cross-reactivity to other *Haemaphysalis* species/sequences. The assay is able to detect a wide range (100,000 fold, 1.984e+7 to 1.984e+2 copies) of *H. longicornis* ITS2 gene copies (Fig. 1). The assay will be useful in determining the species distributions, pathogen prevalence, and conducting ecological studies of *H. longicornis*.

When we applied the assay to the individual submitted samples, we found 37 *H. longicornis* of the 76,004 ticks from July 2006 through December 2020. *Ixodes scapularis*, *D. variabilis*, and *A. americanum* are still dominant human biting tick species in the Eastern United States. These 37 *H. longicornis* were found in 8 states (Connecticut, Massachusetts, Maryland, New Jersey, New York, Pennsylvania, South Carolina, and Virginia) and concentrated in Pennsylvania, New Jersey, and New York (12 ticks in Pennsylvania, 11 in New Jersey, and 8 in New York). Although *H. longicornis* has established in New Jersey, the niche modeling shows that the geographic distribution may be restricted to three sectors—the southeastern United States, the Pacific Northwest, and central and southern Mexico (Raghavan et al. 2019). During 2017–2018, *H. longicornis* was detected in Arkansas, Connecticut, Maryland, New Jersey, New York, North Carolina, Pennsylvania, Virginia, and West Virginia (Beard et al. 2018). The current worldwide geographic distribution of *H. longicornis* showed that this tick can adapt successfully to a wide range of climate regions. The absence of *H. longicornis* from many states might reflect the absence of sampling or failure to discern this species from its congeners.

In the United States, *H. longicornis* was first reported in the literature from a sheep in New Jersey in August 2017 (Rainey et al. 2018). However, our retrospective survey of the passive surveillance archive uncovered an *H. longicornis* that was collected from a human bed in Warren, Somerset County, New Jersey in June 2015, 2 yr earlier than the first published report. This is consistent with the finding that the *H. longicornis* invasion occurred years earlier, for historic samples collected from a deer in West Virginia in 2010 and a dog in New Jersey in 2013 were retrospectively identified as *H. longicornis* (Beard et al. 2018).

DNA of various species of *Anaplasma*, *Babesia*, *Borrelia*, *Ehrlichia*, and *Rickettsia* have been found in *H. longicornis* (Kang et al. 2016). Although all these pathogen groups are endemic in the United States, we did not find any ticks to be infected with *A. phagocytophilum*, *B. burgdorferi sensu lato*, *B. miyamotoi*, *B. mayonii*, *Babesia microti*, and *E. muris*–like agent in this study. This may be due to the limited sample size. However, another study found one of 263 *H. longicornis* ticks tested positive for *B. burgdorferi sensu stricto* (Price et al. 2021). The qPCR-based assay described in this study will be useful for species differentiation and identification, further studies and surveillance of *H. longicornis* to evaluate its geographic distribution and its vector competence and vectorial capacity for various pathogens in the United States.
